# Interoception: A Multi-Sensory Foundation of Participation in Daily Life

**DOI:** 10.3389/fnins.2022.875200

**Published:** 2022-06-09

**Authors:** Carolyn M. Schmitt, Sarah Schoen

**Affiliations:** ^1^Sensory Therapies and Research (STAR) Institute, Centennial, CO, United States; ^2^Rocky Mountain University of Health Professions, Provo, UT, United States

**Keywords:** interoception, sensory integration, sensory processing, sensory processing difficulties, ICF (international classification of functioning disability and health), occupational therapy (OT)

## Abstract

The purpose of this article is to examine evidence that broadens the clinical perspective on interoception as an imperative consideration for individuals with mental health and sensory processing challenges. The central supposition is that interoception is broader than just signals from the viscera. Rather, interoception refers to perceptions of bodily signals and bodily states that construct a subjective representation of the experience. These representations are then utilized for categorizing the sensory attributes and constructing meaning. Thus, this updated conceptualization presents interoception as a complex multidimensional system, with bidirectional features. The interplay between the brain and the body is necessary to maintain homeostasis as well as respond adaptively to the changes in one’s internal and external environment. As a sensory capacity, interoceptive information must be processed and interpreted before it can be integrated into a personal experiential history. Interoception supports both body and mental functions and as such, interoceptive processes support health and wellness by establishing a felt sense of psychological and physiological safety that is foundational to meaningful participation in life. The information presented in this article is central to the pursuit of evidence-based best practices for any professional wishing to integrate consideration of interoception into their clinical practice.

## Introduction

The concept of interoception was first introduced by Nobel Prize winning scientist, Dr. Charles Sherrington. He defined interoception as “sensations from the interior of the body, especially the viscera” (Sherrington, 1906). The past 116 years of scientific advances have expanded knowledge regarding interoceptive processing. Interoception is now defined as the “process of how the nervous system senses, interprets, and integrates signals originating from within the body” (Quigley et al., 2021, p.29). As awareness of interoception grows, the relevancy of interoception to participation in daily life is consequential in determining the future direction of clinical and research fields (Craig, 2015; Tsakiris and Critchley, 2016; Mahler, 2017; Khalsa et al., 2018; Hample et al., 2020). In 2019, the National Institute of Health (NIH) convened a research workshop on the science of interoception and its role in neurological disorders (Chen et al., 2021). One of the priorities identified in that workshop was to grow the collective understanding of how “integrative health approaches may modulate the interoceptive processes and interoceptive clinical outcomes” (U.S. Department of Health and Human Services, n.d.).

Clinicians across many disciplines including mental health and sensory health practitioners are uniquely positioned to consider the impact of interoceptive sensory processing on body functions and structures which effect participation in daily life. This point of view could answer the NIH’s call to action by providing a strong example of the implications of interoception to clinical practice. At its essence, interoception represents the inter-relatedness of sensory, motor, and mental functions enabling perception and participation (Tsakiris and Critchley, 2016). Clinicians from a variety of professions will benefit from expanded knowledge of the contribution of movement and sensation (bottom-up) to mental functions (top-down) and vice versa. In short, movement of the body and interoceptive sensation are the entry points for sense data in bottom-up approaches. Sensory motor experiences are used to inform sense of self, self-regulation, and participation in daily life. In top-down approaches, cognition is utilized to focus attention on the body and make meaning of experience. In this approach, experience changes through understanding whereas in a bottom-up approach, understanding emerges from the experience (Ogden et al., 2006; Ceunen et al., 2016). An approach that includes both top-down and bottom-up strategies reflects a mind-body integrated approach and will guide a common understanding of the impact of interoception on well-being. Interoceptive capacity and its processes support human functioning which is essential to promote meaningful participation in life.

Over the past several years, researchers in multiple fields recognize the contribution of interoception to their area of interest, i.e., trauma, emotion, toileting, hunger and thirst, mental health, the experience of self, decision making, and perception of time (Whitehead and Drescher, 1980; Gallagher, 2000; Drake et al., 2010; Tsakiris et al., 2011; Herbert et al., 2013; Ohira et al., 2013; Suzuki et al., 2013; Herbert and Pollatos, 2014; Pollatos et al., 2014; Barrett and Simmons, 2015; Stevenson et al., 2015; Schreuder et al., 2016; Critchley and Garfinkel, 2017; Grabbe and Miller-Karas, 2018; Quadt et al., 2018; Schaan L. et al., 2019; Schaan V. K. et al., 2019; Zamariola et al., 2019; Mitchell et al., 2020; Quigley et al., 2021). Understanding this proliferation of research is central to the pursuit of evidence-based best practices for any clinician wishing to consider the role interoception may play in impacting function and meaningful participation.

The purpose of this article is to highlight evidence that broadens perspectives on interoception as a critical component of clinical intervention. Interoception does not only refer to perceiving signals from the viscera but rather, interoception refers to perceptions of bodily signals and bodily states, that are generated to construct subjective experience (Fazekas et al., 2020). An expanded definition and interpretation of interoception has been widely considered and the definition presented is based on neuroscience literature. Interoception creates an experiential history within each person, which is utilized for categorizing the sensory attributes of that experience and constructing meaning (Barrett, 2017). Therefore, our aim is to encourage clinicians from every discipline to consider a broader perspective of this multi-dimensional sensory capacity. Interoception is situated within the International Classification of Function (ICF) (World Health Organization [WHO], 2001) conceptual framework of human functioning to illustrate its broad applicability. Viewing interoception as a key component of multi-sensory integration sets the stage for a closer look at the central role interoception plays in body and mental functions that contribute to activity and participation and thus, to overall health and wellness. To illuminate this viewpoint, clinical examples from occupational therapy illustrate how differences in interoceptive sensory processing can impact function and may drive individuals to seek clinical intervention. The final objective is to integrate relevant information and highlight the central role of interoception’s contributions to overall health and wellness.

### Interoception: An Expanded Definition

Recent definitions of interoception embrace the complex, bidirectional interplay between the brain and other organs that is necessary to maintain homeostasis in the moment as well as manage physiological stressors reflective of allostatic processes (Chen et al., 2021; Quigley et al., 2021). Interoceptive sensation originates within the body and travels to the central nervous system. It provides a moment-to-moment physiological representation of the body’s preconscious and conscious internal landscape (Quadt et al., 2018; Tsakiris and dePreester, 2018; Harrison et al., 2019). Interoceptive capacities are used to survey the body and respond to the information based on salience. This information is then communicated to the brain. When the interoceptive information is salient enough that it is deemed important, the brain makes meaning of the incoming sensations (e.g., “My stomach aches, I need to eat”; “I feel lethargic, I don’t feel like playing with my peers”; “My throat hurts, I may be sick”) (Barrett, 2017). If more information is needed to direct action, communication for further sense data is generated *via* descending pathways. While it is the brain that is primarily responsible for interpreting these signals, a notable difference in modern definitions is the inclusion of body regulation through descending pathways (Chen et al., 2021).

Importantly, interoception is not a unitary sensory domain. It is a multidimensional, complex system representing the integration of multiple senses. Sherrington introduced the word interoception connoting interior receptor, which stands in contrast to exteroception, which he recognized as sensation coming from an external source (Sherrington, 1906; Ceunen et al., 2016). Because these sensations were understood to have an origin that was internal or external to the body, early research on body awareness focused on the more easily reproducible exteroceptive signals (Botvinick and Cohen, 1998). We now recognize that those studies were highlighting the power of multi-sensory integration (Tsakiris et al., 2007). A broadened view of interoception as well as advances in neuroscience allow researchers to more fully understand the origin of body awareness and thus demonstrate the important role interoceptive signals play in shaping bodily self-awareness (Quigley et al., 2021) as well as emotion regulation (Price and Hooven, 2018). Contributing to an ordered sense of self is the consideration that the multisensory, interoceptive body summary is a major contributor to regulation, which maintains internal dynamics in balance (Petzschner et al., 2021).

Individuals rely on the automaticity of the sequence of physiological sensation, significance, awareness, and interpretation (Khalsa et al., 2018). When interoceptive signals are processed as anticipated, the result is the ability of that person to trust their body signals (Herbert and Pollatos, 2012; Owens et al., 2018). When interoceptive signals are reliable, by serving to achieve and maintain homeostasis and support overall health and wellness, interoceptive experience establishes a felt sense of safety (Price and Hooven, 2018). In this way, interoception supports the freedom to participate in meaningful activities while trusting one’s body to generate consistent, relevant sensation.

### Interoception Viewed Within a Conceptual Framework of Human Functioning

Interoception is referred to in some literature as an eighth sensory system (Miller et al., 2014; Craig, 2015; Mahler, 2017; Zhou et al., 2021). While this conceptualization draws attention to this critical aspect of human function and is an important step in the evolution of understanding, advances in neuroscience have helped better define the breadth and depth of interoceptive processes. Thus, the expanded definition of interoception addresses its integrative sensory capacity and bidirectional influence and highlights interoception’s role in dynamic processes reflecting both its sensory and regulatory nature.

The relationship between interoceptive sensory processing and meaningful participation in daily life can be conceptualized using the World Health Organization’s International Classification of Functioning, Disability, and Health (ICF) (World Health Organization [WHO], 2001). The ICF framework articulates the inter-relatedness of body functions and structures as well as individual activity and societal participation within health-related human experience. Specifically, this framework considers “multiple dimensions of human functioning synthesizing biological, psychological, social and environmental aspects” (Kostanjsek, 2011, p. 1). Interoceptive sensory processing can be categorized within body functions and structures in the ICF framework. Thus, this conceptualization of interoceptive differences suggests a bidirectional impact on activity performance and participation in life. Overall health and wellness come from the dynamic interplay amongst all these factors.

Applying a health framework with a biopsychosocial lens allows a broader perspective of interoception rather than being linked to a single issue (e.g., emotions) or a specific diagnosis (e.g., autism) (Mahler, 2017; Hample et al., 2020). This lens highlights evidence that interoception underlies many processes of physical and mental human functioning that clinicians across professional fields address in practice (Stucki et al., 2007; Fischer et al., 2017; Khoury et al., 2018). Thus, an expanded approach and the application of a world-recognized framework that considers the multiple elements of this complex multi-dimensional system is warranted. The continuum of health states described in the ICF, specifically the variations in body and mental functioning, is strongly informed and impacted by interoceptive sensory processing. The impacts of this on optimal participation in life will be illustrated in forthcoming clinical examples.

## Interoception as a Dimension of Health

It is important to consider interoception’s contribution to overall health and wellness. Interoceptive capacity acts in the body by allowing basic functions to be automated while one interacts with the external world (Quigley et al., 2021). These signals are essential for regulating many physiological functions (e.g., heart rate, digestion, and body temperature) as well as for psychological experiences ranging from valence to emotion to motivations which acts as a driving force for adaptive behaviors (Khalsa et al., 2018; Chen et al., 2021). It is this interoception-driven adaptive behavior that allows individuals to embody meaningful participation.

Interoception is foundational to the experience and awareness of self and supports one’s ability to trust that their body is relaying and regulating interoceptive sensation in a reliable way (Oldroyd et al., 2019; Chen et al., 2021). Health and wellness come from a sense of agency and a sense of self that contributes to feeling ‘in control’ and mastering interaction with the external world (Di Fabio and Palazzeschi, 2015). A sense of safety comes from the consistency, reliability, and accuracy of how we interpret interoceptive experiences and use those experiences to direct future action (Barrett and Simmons, 2015).

Our body functions are designed to draw context from the environment using sensory data. Through contextual experience, sense data can produce either accommodation and integration or a felt sense of dissimilarity and possibly distress (Meyers-Levy et al., 2010; Köteles, 2021). For example, emotional events may be marked by such somatic reactions as tightening the muscles or increases in heart rate prior to the emotion being brought to consciousness (Barrett, 2017). Individuals count on their body to direct attention to these basic functions so that if we need to flee an unsafe situation or more commonly, void the bladder or eat some food, our bodies comply (Drake et al., 2010; Stevenson et al., 2015). When this process is being perceived or interpreted inaccurately or inconsistently, one’s sense of safety, health and wellness are threatened.

Interoceptive sensory stimuli also assist the brain in creating neural representations of the self and the world (Tsakiris and Critchley, 2016). Importantly, these mental representations underlie perception and drive action. The constant flow of interoceptive stimuli determines the degree of action; memories are stored so that there is a reliable reference to refer to that helps make actions readily available and efficient (Seth et al., 2012). This helps dictate choices and actions of the best ways to respond. For example, when approached by a familiar friend, it is easy to decide to spend time with that friend because the bodily signals convey pleasure and spark memories of past shared experiences. Decisions and actions are then clear about where and how to engage in relationships in the present or future. This ability to direct action appropriate to the context of a situation is also fundamental to one’s felt sense of safety and actual safety.

Interoception is therefore critical for ensuring the stability of the organism in a changing environment as well as the adaptability to external changes. Awareness of the interoceptive body may be fundamental to the unity of the self (Tsakiris and Critchley, 2016). Thus, it appears that interoception has a broader role than once thought that encompasses not only homeostasis and the formation of emotions but how one experiences the self as well as one’s experience of others in social relatedness and meaningful participation. For these reasons interoception is implicated in achieving health, and a pervasive sense of well-being.

## Overview of the Impact of Interoception on Human Functioning

Looking at interoception as a key component of multi-sensory integration and considering interoceptive processes in the preconscious control of one’s physiological state sets the stage for a closer look at the central role interoception plays in body and mental functions that contribute to activity and participation and thus, to overall health and wellness.

### Interoception and Multi-Sensory Integration

For decades, interoception has been integral to the practice of many disciplines from occupational therapists specializing in sensory integration (Ayres, 1972, 1994) to psychologists specializing in emotion (Damasio, 1996, 1999, 2010). These pioneers recognized both somatic and visceral contributions to the integration of interoceptive sensation. Somatic sensory contributions were described as central to the development of the bodily self and physiological alterations to the body’s internal state prior to the formation of an emotion (Damasio, 1996). Similarly, these contributions were recognized as central to the development of one’s body scheme, body map, or body percept, which is the brain’s internal representation of the body (Ayres, 1994). Additionally, internal organ receptors were acknowledged to play a crucial role in regulation of the autonomic nervous system as well as in overall mental and physical health (Ayres, 1994; Damasio and Carvalho, 2013).

Adding to the understanding of interoceptive awareness, which includes this broader conceptualization of somatic and visceral contributions, Khalsa et al. (2018) proposed dividing the concept of interoceptive awareness into components such as attention, detection, magnitude, discrimination, accuracy, insight, and sensibility (Khalsa et al., 2018). Differentiation of these processes are critical to understanding the complex progression involved in the development of body scheme and sense of self. Sensing, determining salience, and interpreting and integrating sensory afferent information from multiple bodily systems is an act of multi-dimensional sensory integration. The multi-dimensional complexity of interoception has also been recognized as an underlying mechanism of many psychiatric disorders (Khalsa et al., 2018; Khoury et al., 2018). This has led to the development of a broader range of interventions based on a focus on bodily sensations, cognitive awareness, and behavioral training (Khoury et al., 2018). Accordingly, interoception should be thought of as a multifaceted system that provides a continual flow of internal sensation that is added to the exteroceptive data in a complex multisensory integration process.

### Interoception as a Preconscious Process

Many conceptualizations of interoception focus largely on one’s level of conscious awareness (Cameron, 2001). In research, for example, laboratory interoceptive measurement tools compel the subject to focus their attention on an interoceptive sensation such as heart rate as a measure of their interoceptive awareness (Sukasilp and Garfinkel, 2022). This type of research favors the cognitive and conscious experience in the study of interoceptive abilities (Garfinkel et al., 2015). Yet, top-down, voluntary focused attention differs from everyday circumstances. Humans typically do not consciously or intentionally monitor their interoceptive sensations. Interoceptive body sensations operate by automatically requiring attention when a shift in body resources demands a person to act to maintain homeostasis (Köteles, 2021). This suggests that interoception operates at the preconscious level wherein a person is primarily unaware of their bodily processes, but these processes can enter consciousness when top-down attentional resources are directed to the process (Balconi et al., 2017).

The idea that interoception is not limited to conscious perception, but extends to and primarily functions as preconscious perception, is an important distinction (Damasio and Carvalho, 2013; Sukasilp and Garfinkel, 2022). Quadt et al. (2018) consider interoception an overarching term for several processes including (a) afferent sensory signaling, (b) neural encoding, representation, and integration of the information concerning our internal body state, (c) how this information influences other perceptions, cognitions, and behaviors, and (d) the consciously accessible physical sensations and feelings generated by these representations. Three of the four processes are preconscious. This differentiation of interoceptive processes is important to clinicians because interoceptive processes are implicated in the preconscious control of one’s physiological state, which forms the basis for emotions, behavior, and cognition (Quadt et al., 2018). Over-emphasizing the conscious mind over the sensing body obscures the wholistic consideration of the mind-body connection and diminishes the importance of preconscious interoceptive processes. Damasio and Carvalho (2013) capture the nuance of sensory perturbations (experienced when reality does not match mental predictions) and their role in eliciting and informing cognitive interoceptive attention. A dynamic relationship exists in the contribution of physiological body function to mental functioning (e.g., higher-level cognition, attention, memory, affect, perception, etc.) and vice versa (one’s mental state influences the body’s physiological state). This recognition of the dynamic, preconscious features of interoceptive sensation will serve clinicians in establishing bottom-up intervention strategies to improve interoceptive abilities.

### The Contribution of Interoception to Body Functions and Structures Affecting Participation

Within the ICF framework, the list of Body Functions and Structures are representative of the two branches of the interoceptive system, the visceral system reflected by internal organ functions (e.g., cardiovascular, respiratory, genitourinary) and the somatic system represented by neuromusculoskeletal and skin structures (World Health Organization [WHO], 2001). Narrow conceptualizations focused solely on the viscera as the primary contributor to interoceptive sensation (Sherrington, 1906). Advances in neuroscience have promoted the inclusion of the somatic systems to the interoceptive domain (Chen et al., 2021). Both visceral and somatic signals originate from the body and are relayed as afferent sensory data (Craig, 2015; Ondobaka et al., 2017; Chen et al., 2021). Once the sensory information reaches the brain, it is integrated with other sense data to represent the physiological condition of the body (Ceunen et al., 2016; Khoury et al., 2018). Linking visceral and somatic bodily sensations with perception supports the development and experience of one’s selfhood (Khalsa et al., 2018; Marshall et al., 2018; Tsakiris and dePreester, 2018).

Activity performance and participation in daily life are closely interrelated with intrinsic factors such as sense of self (Imms et al., 2015). One’s sense of self or the way one perceives one’s body from the inside interacts with and enhances the way one perceives one’s body and other people’s bodies externally (Ondobaka et al., 2017). These findings underscore the importance of considering visceral and somatic interoceptive sensation and their role in multisensory processing as it allows individuals to experience their bodies as their own and thus impacts their functional participation (Tsakiris et al., 2011; Suzuki et al., 2013). The following section will present data on the role visceral and somatic sensory afference and illustrate how interoceptive sensory differences impact function and affect meaningful participation.

#### Visceral Sensory Afference

Tsakiris and dePreester (2018) use the term visceral afference to refer to internal sensory information that is processed within the viscera of the interoceptive system. The authors also include olfactory and gustatory receptors (chemoreceptors) in the visceral system while Craig (2015) indicates that pain and temperature receptors are considered interoceptive (e.g., stomachache) or exteroceptive (e.g., burn) based on the source of the sensory experience. Chen et al. (2021) further delineate these internal sensory signals into three types: biochemical, mechanical forces, and thermal or electromagnetic signals. Though the receptor characteristics and neural pathways vary, an understanding of the central role of afferent visceral signaling to an individual’s physiological state contributes to clinical considerations of issues such as digestive functions, including discrimination of hunger/satiety, or gastrointestinal and urinary tract functions, such as bowel and bladder voiding.

##### Clinical Example of Visceral Sensory Afference

This clinical example highlights the role of visceral-based factors in the treatment of a teenager with differences in sensory processing and integration. Evelyn is a 15-year-old girl who first received occupational therapy services at age eight. At that time, standardized testing revealed deficits in sensory discrimination and sensory-motor abilities, supported by parent rating of dysfunction in Evelyn’s reactivity to sensory experiences. She returns seven years later seeking occupational therapy due to recent weight gain and the challenges associated with attending high school while still on a toileting schedule. A review of systems indicates no medical concerns about the function of her gastrointestinal or urinary tract function. In gathering intake information, Evelyn reports eating without ever perceiving feeling satisfied/full (lack of awareness/sensory under-responsivity) and being unable to discern (sensory discrimination) the need to empty her bladder or bowel. Research suggests that the forebrain mediates the transition between urine storage and urine voiding (Drake et al., 2010). This process is dependent on visceral afferent interoceptive sensory information from the bladder to guide the timing of the transition between the two phases (storage and voiding). Regarding Evelyn’s weight gain and reported challenges feeling satiety, Herbert et al. (2013) and Herbert and Pollatos (2014) found evidence for reduced interoceptive sensitivity in overweight and obese individuals by identifying challenges in the detection of bodily changes associated with feeling full. The threshold required for Evelyn to register bladder and stomach fullness is high. Her bladder and stomach must be very full for her interoceptive sensation to rise to a level of salience so that her cognitive attention guides her action to void her bladder or stop eating. In a recent study, Mitchell et al. (2020) found a significant positive correlation between altered interoceptive awareness and hypo/hyper-reactivity to sensation. Utilizing a sensory integrative intervention framework to address sensory under responsivity would focus on enhanced stimulation of bodily sensations, accompanied by, or linked to cognitive awareness. The intervention prioritizes both bottom-up sensing and top-down regulation, engaging bi-directional sensory data (Chen et al., 2021). This is accomplished by offering opportunities for multi-sensory experiences and exploration through active participation in age appropriate sensory-motor experiences (e.g., dance, balance work, exploring sensory strategies that alter arousal through touch, smell, movement or sound), which is both internally motivating and calibrated to promote success (Ayres, 1994; Caçola, 2016; Miller et al., 2018). In addition, cognitive strategies such as asking questions about her sensation and using body mapping to locate those sensations direct Evelyn’s attention to her bodily experiences. Simply put, clients like Evelyn are supported when the objective of intervention is to maximize the interplay between brain-body processes and enhance bodily signals allowing for the interoceptive sensation to be recognized as meaningful. The goal is to support Evelyn’s pursuit of meaningful and successful participation at school and home through improved interpretation of visceral interoceptive sensory processing.

#### Somatic Sensory Afference

Somatic afference refers to the activation of receptors in the somatic system of muscles and joints. These are senses that help individuals gather information about one’s internal and external world and one’s relationship to it (Quadt et al., 2018). Considering somatic afference is an important feature many clinical interventions frequently use for psychiatric disorders as well as motor disorders affecting participation in daily life (Khalsa et al., 2018; Khoury et al., 2018).

##### Clinical Example of Somatic Sensory Afference

Somatic interoception can be best understood through another clinical example. Emma is a ten-year-old referred to occupational therapy due to concerns regarding performance in group athletics and social interaction with peers, including physical education classes at school, organized sports teams, and playing with peers during recess. She appears clumsy, runs into her peers, is not goal-directed in her actions, and is unable to learn new games easily. Standardized testing revealed significant challenges in upper limb coordination, bilateral coordination, and balance as well as dysfunction in sensory discrimination in vestibular and proprioceptive processing. Difficulty was noted interpreting information from her muscles and joints and vision while simultaneously moving through space. She registers the input but is unable to interpret the details necessary to generate smooth, coordinated motor efforts. The result is poorly refined movement which impacts her ability to participate fully in age-appropriate and meaningful play. Administration of the Multidimensional Assessment of Interoceptive Awareness, Youth Version (MAIA-Y) (Jones et al., 2020) reveals below average scores in the categories of Noticing (awareness of body sensations) and Body Listening (ability to attend to bodily sensation for psychological insight). This assessment assists in clarifying the origin or generation of the sensory signaling as a function of input from multiple visceral and somatic sources across temporal domains. Interoception has an undeniable role in differentiating these stimuli and in integrating these sensations (Craig, 2003, 2009, 2015). This example captures the complexity of interoception’s contributions to body awareness, embodiment, and sense of self. Gallagher (2000) elaborates on this association by recognizing that the bodily sense of agency and ownership for motor action is based on sensation, which precedes action. Once the process is conscious, the brain generates intention and drives action.

The sensation referred to here is interoceptive in nature and contributes to motor coordination in collaboration with stored representations of past motor experience. Motor experience was emphasized during Emma’s intervention, with multisensory feedback to enhance the discrimination of interoceptive sensation and support the refinement of motor coordination in time and space. The focus on somatic awareness in intervention incorporated repetitive, rhythmical movements within the context of body-based activities in the sensory gym. Additionally, slow controlled movements of varying speed and timing were combined with examples provided through mirroring activities/movements. This type of sensory and motor exploration was encouraged so that sensations would be approached with interest, variety, and curiosity (Ogden and Minton, 2000; Hricko, 2011; Levine, 2018; Goldstein, 2021).

### The Contribution of Interoception to Mental Functions Affecting Participation

Interoception is also foundational to the mental functions relevant to wellbeing and health and makes significant contributions to the attainment and maintenance of these mental functions. Specific mental functions relevant to clinical consideration of interoception include affective, cognitive, and perceptual components. Affective neuroscience research frequently adopts predictive processing models which explore how predictions and prediction errors generate subjective experience and inform neural representations of the world (Barrett, 2017; McTighe and Willis, 2019). The formation of a neural representation of the world demonstrates the intersection of mental functions and interoception (Quigley et al., 2021).

#### The Predictive Processing Framework

The predictive processing framework clarifies how interoceptive signals affect mental function. Sensory signals from the body are processed by the central nervous system (Barrett and Simmons, 2015; Quadt et al., 2018). The brain compares the sensation to past experience to formulate a prediction or hypothesis that can be tested against incoming sensory signals. If ambiguity or an error in that prediction exists, there are three options: (a) transmit the error back along cortical connections and modify the prediction; (b) generate an action/response/movement to match the predicted sensations; or (c) regulate the attention to the incoming sensory signals (Barrett and Simmons, 2015). The brain’s job is to utilize this information for higher-level cognitive functions as well as emotion, attention, memory, thought, and experience of self and time (Pollatos and Schandry, 2008; Matthias et al., 2009; Werner et al., 2010; Pollatos et al., 2014; Barrett and Simmons, 2015; Critchley and Garfinkel, 2017). The contribution of interoception to mental function entails attending, interpreting, and prioritizing the stimuli that will promote the body/brain’s ability to achieve and maintain internal order for optimal health, safety, and survival.

The forebrain uses the above process to form a concept based on available sensory input (McTighe and Willis, 2019). The concept formation is important to efficient use of the brain’s power in the moment as well as in the future. In grouping some things and separating others, the brain becomes more efficient and better able to interpret the meaning of incoming sensory inputs. The brain uses this process to make meaning of sensations from both outside as well as inside the body. Past experiences are organized into concepts that are used to guide actions and give meaning to incoming sensory signals (Barrett, 2017; Ondobaka et al., 2017).

#### Interoception and Emotion

It has long been held that emotion is inextricably linked to unique bodily states (James, 1994). Emotions arise through the interaction of descending bodily predictions and ascending prediction errors (Critchley and Garfinkel, 2017). Schreuder et al. (2016) note that harmonious multi-sensory stimuli enhance emotional, cognitive, and behavioral responses while disparate stimuli negatively impact emotion and adaptive behavioral responses. The congruent or incongruent processing of multi-sensory stimuli may be a contributor to or underlie behavior regulation. And while behavior is not controlled by emotion, sensory input plays an important role in how one feels, how one understands that feeling, and thus how one behaves (Herbert and Pollatos, 2012; Schreuder et al., 2016). Quadt et al.’s (2018) interoceptive predictive processing framework explores these brain-body interactions, to the extent that prediction errors raise interoceptive sensations to the level of cognitive awareness and thus may contribute to both physical and mental health concerns presenting as emotional impairment.

##### Clinical Example of Emotion

This example highlights relevant mental and emotional-based factors practitioners may encounter in children with sensory integration and processing differences. Justin is an 8-year-old boy who is accompanied to occupational therapy by his parents who express concern for his emotional volatility at home and school causing significant relational strain and reduced social participation. Standardized testing revealed developmental dyspraxia resulting in problems in organization of movement and motor planning difficulties. Justin is described as escalating from “zero to 100” in a matter of moments and across a variety of situations/contexts. Emotion regulation is frequently impacted in children who experience dyspraxia due to the high levels of frustration they encounter when attempting to participate in everyday activities. Parented conscientiously, Justin has been taught plentiful top-down, cognitive strategies. However, Justin is frequently not able to access or apply these strategies as he is often living in a state of generalized stress and dysregulation. Applying the interoceptive predictive processing framework to the clinician’s clinical reasoning, it is hypothesized that Justin’s system is encountering repeated unexpected sensory signals, eliciting prediction errors, and causing stress that exceeds his homeostatic threshold. Beyond this homeostatic threshold, activation of his stress response means executive functions and cognitive regulation strategies are unavailable to him (Dang, 2016). This reflects a shift of interoceptive resources away from homeostasis and toward a fight or flight or freeze reaction (Porges, 2009). Justin often exhibits a fight response disrupting his home and school environments. When the actual experience does not match Justin’s predictions, a prediction error arises. This prediction error disproportionately affects Justin’s narrow range of emotion regulation (Tsakiris and Critchley, 2016). Intervention focusing on improved sensory discrimination will support progress toward improved praxis which will promote emotion regulation through a reduction in frustration. Prioritizing activities that are not so easy they are not beneficial but not too challenging to be unachievable will promote a reduction in prediction errors and aim to widen the amount of time Justin spends in desirable regulatory states. Using this approach, clinical intervention shifts the focus away from an over-reliance on cognitive strategies to focus on non-verbal, preconscious bodily responses and movement activities that are adaptive to the situational challenge. Justin was not functioning adaptively within his home or school environment. An intervention designed to help Justin achieve and maintain a regulated state from which to express appropriate responses to environmental demands promoted participation in both meaningful tasks and relationships.

#### Other Mental Functions

Evidence exists that interoceptive sensory processing plays a crucial role in higher-level cognitive functions, attention, memory, and the experience of self and time (Pollatos and Schandry, 2008; Matthias et al., 2009; Werner et al., 2010; Pollatos et al., 2014; Barrett and Simmons, 2015; Critchley and Garfinkel, 2017). Craig (2009) and Critchley (2009) have both identified the insula as the recipient of visceral and somatosensory input. As such, the insula is thought to play a significant role in the integration of interoceptive sensation contributing to the neural representation of bodily and mental states (Damasio, 1999). Ohira et al. (2013) explored the neural basis of decision-making looking primarily at the anterior portion of the insula. They found that the role of interoception in bodily states had implications for decision-making due to the strategic location of the insula as a hub linking visceral and somatic input with the prefrontal and limbic regions (Craig, 2009; Critchley, 2009). Matthias et al. (2009) investigated interactions between interoceptive awareness and measures of attention finding that interoceptive perception may moderate visual processing and utilize brain processing resources of the self-focused attention system.

Multiple researchers document a substantial role of interoceptive stimuli, particularly visceral feedback, in implicit memory processes (Werner et al., 2010). Pollatos and Schandry (2008) investigated the relationship between interoceptive awareness and the conscious processing and memory of emotional information. High levels of interoceptive awareness correlated with stronger responses to positive or negative stimuli, suggesting that interoceptive processing of emotionally heightened stimuli improves our ability to form memories of that stimuli. Conversely, when stimuli trigger the fight, flight, or freeze response, the experience is less likely to be encoded in explicit memory (Siegel, 2008). Applying this to individuals who experience sensory integration and processing differences resulting in high levels of stress and heightened negative emotions suggests their ability to establish explicit memories may be negatively impacted.

##### Clinical Example of Other Mental Functions

Scarlett is a 16-year-old girl who seeks occupational therapy because of concerns related to sensory over-responsiveness to sound and touch as well as difficulty with emotion regulation. Over responsivity was reported on self-report and parent-report measures as well as during standardized testing in the clinic. Over-responsiveness is characterized by responses to sensory stimuli that are faster, longer, or more intense than what would be expected with typical sensory responsivity (Miller et al., 2007). Despite her extremely high IQ, her sensory differences result in challenges in self-expression particularly as it relates to decision making and problem solving. Scarlett reports living in a constant state of stress. Sounds produce a heightened experience that triggers the fight, flight, or freeze response, and are likely not being encoded in explicit memory. She indicates feeling confused by sensory signals from a young age and constantly feeling overwhelmed by auditory and tactile input. She is intellectually gifted but reports struggling to manage the incongruence between her mind and bodily experiences. Decision-making is particularly impacted. For example, in multisensory environments like a restaurant, when faced with ordering food from a menu, 16-year-old Scarlett completely freezes due to her over-responsivity. She is reliant on her family members to order for her. Her concerns reflect a challenge in executive functions and body-based regulation resulting in frustration and inaction. For Scarlett, her body’s protective response is funneling interoceptive resources away from homeostasis and toward a freeze reaction (Porges, 2009). This compensatory strategy affects the encoding of memories necessary for problem solving and decision-making. Scarlett’s experience highlights the relevance of interoceptive sensory inputs’ cascading effect on complex social behaviors including the demand for executive function (Adolfi et al., 2017). Knowledge derived from research establishes that instances of homeostatic perturbation offer a period of time in which the conscious perception of interoception can be elicited (Critchley et al., 2004). Scarlett’s clinician utilizes this as a tool for intervention by incorporating opportunities for physiological exertion that require a homeostatic shift or tax the system in a way that the system can respond with a rebalancing or return to homeostasis. In response, timely use of cognitive strategies such as attending to, and labeling bodily sensations is advantageous. The goal of this strategy is to generate sensory stimuli through movement of the body and then engage the forebrain by making interoceptive sensory stimuli conscious, thus formulating awareness and driving behavioral responses. This example can guide practitioners to incorporate bottom-up and body-based interventions along with cognitive strategies to drive adaptive behavioral responses but most importantly to work toward body and mind congruence which offers a felt sense of safety and promotes purposeful engagement in life.

## Conclusion

The proliferation of evidence on the pervasive influence of interoception offers a timely opportunity. Future clinical and research endeavors can be carefully considered to offer an expansion of knowledge as well as a new dimension to the science underlying intervention. The knowledge base provided from multiple fields provides a powerful and undeniable insight into the role of interoception in the interaction between body, brain, and mind. To further the knowledge, there is a need for the development of interoceptive assessment tools. These tools need to document the association between interoception and engagement in meaningful participation. The ability to assess interoception in support of meaningful participation will guide clinicians in understanding interoception’s role in the underlying sensory processes. Where differences in interoceptive processing are captured, these differences can be related to indicators of diminished function. Drawing from this, therapeutic intervention which targets interoception could prove to support the individual’s sensory health and wellness.

In support of the evidence related to interoceptive capacity, interventions that are developed need to support both top-down and bottom-up foundational strategies (Khoury et al., 2018). The evidence generated must test intervention procedures across the lifespan and encompass a variety of clinical populations. Interventions addressing body-based activities, bodily sensations, and body awareness, for example, could improve interoceptive accuracy, interoceptive regulation, interoceptive self-efficacy, and support remediation of many clinical disorders impacting function (Fischer et al., 2017; Khoury et al., 2018). The anticipated improvement in the ability to detect and distinguish interoceptive signals may prove helpful in contextualizing this sensation for improved meaning-making which drives a felt sense of safety and promotes well-being. Such efforts will grow the collective understanding of how evidence-based practice impacting interoception can support meaningful participation in life through improved sensory health and wellness.

## Data Availability Statement

The original contributions presented in the study are included in the article/supplementary material, further inquiries can be directed to the corresponding author/s.

## Author Contributions

All authors listed have made a substantial, direct, and intellectual contribution to the work, and approved it for publication.

## Conflict of Interest

The authors declare that the research was conducted in the absence of any commercial or financial relationships that could be construed as a potential conflict of interest.

## Publisher’s Note

All claims expressed in this article are solely those of the authors and do not necessarily represent those of their affiliated organizations, or those of the publisher, the editors and the reviewers. Any product that may be evaluated in this article, or claim that may be made by its manufacturer, is not guaranteed or endorsed by the publisher.
